# Brillouin Dynamic Gratings—A Practical Form of Brillouin Enhanced Four Wave Mixing in Waveguides: The First Decade and Beyond

**DOI:** 10.3390/s18092863

**Published:** 2018-08-30

**Authors:** Arik Bergman, Moshe Tur

**Affiliations:** 1 Faculty of Engineering and Institute for Nano-Technology and Advanced Materials (BINA), Bar-Ilan University, Ramat-Gan 5290002, Israel; 2 School of Electrical Engineering, Tel-Aviv University, Tel-Aviv 6997801, Israel; tur@post.tau.ac.il

**Keywords:** dynamic gratings, stimulated Brillouin scattering, optomechanics, optical data processing, fiber optics sensors

## Abstract

Brillouin-Enhanced Four-Wave-Mixing techniques, which couple four optical beams through Brillouin nonlinearity, have gained popularity in the 1980’s largely owing to their phase conjugation properties. Experiments were mainly conducted in liquid cells. The interest in Brillouin-Enhanced Four-Wave-Mixing has reawakened in the 2000’s, following the quest for dynamically reconfigurable gratings in optical fibers. Termed Brillouin Dynamic Grating this time around, it is, in fact, an acoustic wave, optically generated by stimulated Brillouin scattering process between two pump waves. The acoustic wave either carries the coherent information encoded by the pump beams, or in the case of sensing applications, its properties are determined by the environmental parameters. This information, in turn, is imparted to the third phase-matched optical probe wave through the elasto-optic effect. Over the last decade, this mechanism allowed for the realization of many all-optical signal processing functions and has proven instrumental in distributed sensing applications. This paper describes the basics, as well as the state of the art, of BDG-based applications in optical fibers. It also surveys the efforts being done to carry over these concepts to the photonic chip level.

## 1. Introduction

In Brillouin-Enhanced Four-Wave-Mixing (BE-FWM) [1,2], the four optical waves interact with each other through a material density wave excited through a combination of a Stimulated Brillouin Scattering (SBS) process, that generates a moving optical intensity wave and the phenomenon of electrostriction, where the optical intensity wave gives rise to a corresponding density wave [3]. From the late 70’s until the early 90’s, BE-FWM in bulk media was the subject of extensive research, leading to applications such as optical phase conjugation, beam combining and optical path selection [4,5,6]. Bayvel and Giles were the first to demonstrate the BE-FWM process in optical fibers in 1990 [7]. However, it is not until 2008 that this process regained interest, when Song et al. proposed using it as a sort of dynamically reconfigurable grating, coining the term Brillouin Dynamic Grating (BDG) [8]. It has since been shown to be a particularly flexible technique for the realization of all-optical calculus [9], microwave photonic reconfigurable filters [10], tunable optical delay lines [11,12], coherent acousto-optic memory and pulse compression [13,14], frequency combs [15], ultrahigh-resolution optical spectrometry [16], distributed measurement of birefringence and intermodal beat length [17,18], and last but not the least, high spatial resolution and distributed, static and dynamic sensing [19,20,21,22,23,24]. Recent years have also evidenced a growing consolidation of fiber and integrated devices technologies, creating new research challenges and opportunities.

The BDG is, in fact, a special case of BE-FWM, where the two optical pump waves determine the spatio-temporal form of the acoustic wave, and by choosing the power of the probe wave to be sufficiently low, the problem at hand becomes that of diffraction from a moving fiber Bragg grating [8]. The peak efficiency of this process is achieved when phase-matching conditions for both the generation of the acoustic field via SBS process and the reflection of the incoming probe wave from the induced BDG are met. Normally, the acoustic waves span a frequency range from several GHz [25] to several hundreds of GHz [26].

In order to efficiently separate the writing and reading optical waves, the interaction is tailored such that they occupy orthogonal modes of propagation (e.g., polarization states, spatial modes, entirely separate waveguides, etc.). In the vast majority of practical cases, the writing optical waves satisfy the phase-matching condition in a counter-propagating orientation only, allowing for straightforward localization of the grating [27,28,29]. Coupling between co-propagating pumps, peculiar to intermodal SBS [30], obstructs possibilities of confining the grating along the waveguide, precluding some BDG applications.

When the BDG plays the role of an all-optical, reconfigurable, signal processing circuit, its key performance specifications are accuracy, optical signal-to-noise ratio, bandwidth, dynamic range, and other application-unique requirements. On the other hand, when BDG functions as a distributed reflectometer/sensor, its performance can be evaluated based on sensitivity, spatial resolution, range, sampling rate, precision, cross-sensitivity and so forth. Other common design rules may include low-power operation, integration, complexity, cost and so forth.

In this review, we revisit the principle of operation (Section 2) and survey the platforms which have accommodated BDG interactions thus far (Section 3). We also present the materials choice and geometry considerations for future BDG designs (Section 4). Then we turn to detailed description of the methods for BDG generation and readout (Section 5). Section 6 provides an overview of the state of the art of BDG applications. Finally, conclusions are drawn in Section 7.

## 2. Principle of Operation

**Figure 1:**
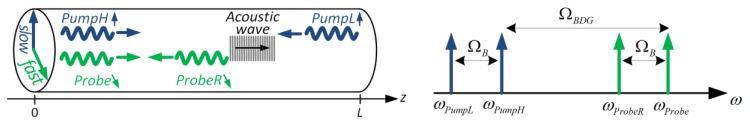
Brillouin dynamic grating principle of operation in polarization maintaining fibers.

Here we present the BDG principle of operation for the special case where orthogonal, linearly polarized pump and probe waves are fed into the principal axes of a Polarization Maintaining Fiber (PMF). The interference of the two co-polarized, counter-propagating pump waves (*PumpH* and *PumpL*) creates a moving optical intensity pattern of angular frequency $\Omega =\omega_{PumpH}-\omega_{PumpL}\;(>0)$ and wavenumber $q=k_{PumpH}+k_{PumpL}$ (the ω’s and k’s obey their respective optical dispersion relation: $\omega =kc/n_{slow}$, where c is the light velocity in vacuum, and $n_{slow}$ is the refractive index along the fiber slow axis). Owing to the phenomenon of electrostriction (the tendency of materials to become denser in regions of high optical intensity [31]), the moving intensity wave induces a corresponding density wave, which moves in the same direction as the higher frequency pump wave, *PumpH*. The generation of this longitudinal density wave is not efficient unless Ω and q obey the dispersion relation of acoustic waves in the fiber: $\Omega =qV_{a}$, where $V_{a}$ is the velocity of longitudinal acoustic waves in the fiber. This phase-matching condition ensures maximum growth of the acoustic wave, provided that the frequency of the density disturbance, Ω, is equal to the slow axis Brillouin Frequency Shift (BFS): $\Omega =\Omega_{B}\equiv 2n_{slow}V_{a}\omega_{PumpH}/c$. Via the elasto-optic effect, this induced longitudinal acoustic wave is actually a moving refractive index (Bragg) grating, which can back-reflect an orthogonally polarized *Probe* wave into a backward-propagating fourth wave, *ProbeR*. The angular frequency of the reflected wave, $\omega_{ProbeR}$, is dictated by the angular frequency of the receding grating, Ω, namely: $\omega_{ProbeR}=\omega_{Probe}-\Omega$. The reflection reaches its highest efficiency when the wavenumbers of the signals involved in the process satisfy the phase matching condition: $\Delta k=k_{PumpH}+k_{PumpL}-k_{Probe}-k_{ProbeR}=0$. This condition can be further simplified and approximated as a condition on $\omega_{Probe}$: $\omega_{Probe}-\omega_{PumpH}=\Omega_{BDG}\equiv \omega_{PumpH}\left(n_{slow}-n_{fast}\right)/n\;$ ($n_{fast}<n_{slow}$ is the refractive index along the fiber fast axis and $n=\left(n_{slow}+n_{fast}\right)/2$ is the mean refractive index). The refractive indices difference $n_{slow}-n_{fast}\equiv \Delta n$ is a common definition of birefringence [31]. The BDG principle of operation is illustrated in Figure 1.

When the initial powers of writing optical waves and Brillouin amplification are both weak and for short interaction length L, *PumpH* depletion and *PumpL* amplification, as well as their linear losses, can be neglected. Furthermore, under weak-coupling conditions, which BDG certainly satisfies [32], the *Probe* also remains undepleted. We also assume that the hypersonic phonons are strongly damped and thus propagate only over very short distances before being absorbed [31]. When the aforementioned conditions hold, and we further assume that the amplitudes vary slowly in space and time, the governing differential equations of BDG interaction can be analytically solved to give an approximate expression for the steady-state reflection spectrum of a continuous-wave (CW) *Probe* [33]:

(1) $$\begin{array}{ll} \;R\left({\Delta \Omega }_{B},{\Delta \Omega }_{BDG}\right) & {\;}_{\sim }^{\propto }\;g_{B}^{2}I_{PumpH}I_{PumpL}L^{2}\;\frac{{\left(\Gamma_{B}/2\right)}^{2}}{{\left({\Delta \Omega }_{B}\right)}^{2}+{\left(\Gamma_{B}/2\right)}^{2}}\;{\sin c}^{2}\left[\left({\Delta \Omega }_{BDG}n/c\right)L\right]\\ & \equiv g_{B}^{2}I_{PumpH\;}I_{PumpL}L^{2}\;R_{BGS}\left({\Delta \Omega }_{B}\right)R_{BDG}\left({\Delta \Omega }_{BDG}\right). \end{array}$$

Here, ${\Delta \Omega }_{B}\equiv \Omega -\Omega_{B}$ measures the deviation from phase-matching condition for the creation of the acoustic field, while ${\Delta \Omega }_{BDG}\equiv \left(\omega_{Probe}-\omega_{PumpH}\right)-\Omega_{BDG}\approx -\Delta k\cdot c/2n$ is a measure of the phase matching between the induced acoustic field and the incoming *Probe* wave. $I_{PumpH}$ and $I_{PumpL}$ are the intensities of *PumpH* and *PumpL*, $g_{B}$ is the Brillouin gain, and $\Gamma_{B}$ is the acoustic decay rate [31].

Equation (1) expresses the separable nature of the creation of the acoustic wave and its interrogation. We intentionally chose to present the result as a product of two physically meaningful factors: a Lorentzian-shaped Brillouin Gain Spectrum (BGS), $R_{BGS}\left({\Delta \Omega }_{B}\right)$, and the reflection spectrum of a moving Bragg grating of length L, $R_{BDG}\left({\Delta \Omega }_{BDG}\right)$, both normalized. The BGS linewidth is governed by the longitudinal phonon lifetime $\tau_{A}=1/\Gamma_{B}$ (typically ~30 MHz in silica). The weak Bragg grating bandwidth is inversely proportional to the interaction length: ∝c/nL. A remarkably narrowband BDG reflection of 0.5 MHz was achieved in a 400 m Single-Mode Fiber (SMF) [16].

To facilitate low-power operation of BDG, large Brillouin gains are essential. The Brillouin gain in optical fibers, the leading quadratic factor in Equation (1), predominantly depends on intrinsic nonlinear properties of the waveguide material, and less on the acousto-optic effective area, found in the denominator of $g_{B}$ [34]. In microscale waveguides, we rely on scalar SBS treatment to evaluate the Brillouin gain [35]. We assume that electrostriction couples only transverse-electric waves to longitudinal elastic waves through the elasto-optic tensor. Within the limits of this assumption, the acousto-optic effective area is calculated by integrating the overlap between the radial profiles of the optical and acoustic modes. To sum up, optical fibers lack the geometric degrees of freedom necessary to augment the photon-phonon coupling strength. This paradigm, however, breaks down when the size of the waveguide shrinks. Effects of boundary-induced nonlinearities and radiation pressure kick in, both of which having tremendous impact on photon-phonon coupling when waveguides approach subwavelength scales [36]. Material solutions are also being investigated [37], paving the way to BDG processes with ever smaller translational dimensions.

## 3. Available Platforms

To date, the workhorse of BDG interactions was the PMF. Despite its relatively large birefringence non-uniformity, the ability to separate the probe reflection both polarizationally and spectrally ($\Omega_{BDG}$ is proportional to $n_{slow}-n_{fast}$), combined with commercial availability, made PMF very attractive for most BDG applications.

Sacrificing the ability to separate the probe reflection spectrally, yet benefiting from its small birefringence non-uniformity, SMF can be a cheaper alternative to PMF [38]. However, without special handling, such as winding the SMF around a bobbin [16,38], the random evolution of polarization states in the fiber makes the usage of SMFs, longer than a few meters, impractical. A partial circumvention of this drawback makes use of fast optical switching, albeit with limited performance [39,40]. A multitude of acoustic modes supported by the single-mode dispersion-shifted fiber (DSF) geometry, offers a frequency-shifted interrogation of BDG with somewhat limited power efficiency [41]. This last demonstration is not a BE-FWM in the strict sense, but rather an intriguing manifestation of a complex Brillouin interaction involving multiple optical and acoustic waves.

Modal birefringence in Few-Mode Fibers (FMFs) can also be instrumental in facilitating BDG interactions [42]. The writing and reading optical waves in hitherto experimentally-validated FMF-BDG configurations, occupy orthogonal spatial modes, which results in reduced overlap integral and subsequently in a weaker acousto-optic coupling. The main incentive for employing those FMFs was, therefore, their mechanical properties rather than the optical ones. For instance, an elliptical core was found to have higher birefringence sensitivity to transverse load and lower temperature crosstalk compared to PMF, at the expense of poorer signal-to-noise ratio [21]. BDG generation through intermodal backward SBS, utilizing different types of acoustic modes, can potentially exhibit appreciable strength in a range of fiber and integrated waveguide geometries [43].

Glass Photonic Crystal Fibers (PCFs) guide light by means of a lattice of hollow micro-channels running axially along its length [44]. The large refractive index difference between air and glass allows much tighter confinement of light than is possible in all-solid glass optical fibers. The hard glass-air interfaces of PCFs also couple acoustic longitudinal and shear waves together, and the resulting acoustic modes contain proportions of both dilatational and shear strain. The presence of the latter reduces the effective refractive index perturbation [45]. Despite the high optical power density compared with standard all-solid silica fibers, the overall efficiency of BDG generation in PCFs is significantly smaller. Nevertheless, its porous structure is more susceptible to deformation and, for instance, advantageous in measuring hydrostatic pressure [22].

The first planar platform to accommodate BDG interaction was a chalcogenide (As_2_S_3_) rib waveguide [46]. The effect was 2–3 orders of magnitude stronger than in silica fiber owing to the pronounced intrinsic nonlinearity of the chalcogenide glass, small effective mode area, and strong confinement of acoustic and optical modes in the rib structure. The strong birefringence of the waveguide enabled writing a BDG in linear-*x* polarization and characterizing it using a CW probe in the linear-*y* polarization. The broadening of the BDG resonance, $R_{BDG}\left({\Delta \Omega }_{BDG}\right)$, was attributed to the non-uniformity of the waveguide.

Another promising gateway to integrated BDG-enabled all-optical signal processing is the silicon-on-insulator (SOI) platform. However, nonlinear propagation in standard silicon waveguides at telecommunication wavelengths is restricted by two-photon absorption (TPA) and free-carrier absorption (FCA). Moreover, the SOI platform lacks acoustical confinement in the silicon core, resulting in acoustic field radiation into the silica substrate [47]. Sophisticated solutions such as nano-waveguides suspended by a series of nanoscale tethers [48] or supported by a pillar [49], that simultaneously guide light and sound waves, were recently proposed, demonstrating Brillouin gains four to five orders of magnitude larger than in silica fibers.

BDG-like operation in those devices is realized by fabricating two silicon waveguides that share the same suspended membrane [50]. Membrane vibrations are generated through intermodal SBS of the two co-propagating, symmetric and anti-symmetric optical modes in the “write” waveguide. The driven elastic wave time-modulates the refractive index across the membrane, mediating the intermodal coupling in the “read” waveguide. Thus far, this concept was used to demonstrate optical isolation, namely, probe light which propagates in the opposite direction does not experience a phase-matched scattering process with the acoustic wave, and thus propagates through the waveguide unaffected.

## 4. Materials Choice and Geometry Considerations

SBS is among the principal phenomena restricting continued scaling of high power-per-unit-bandwidth systems. While considerable efforts have been made in recent years to suppress the Brillouin scattering using material-based approach [51], the same principles can be employed to design a glass composition favouring Brillouin scattering. Beneficial material physical properties for the enhancement of SBS include [35]: (1) a small acoustic velocity, $V_{a}$; (2) relatively high refractive index; (3) relatively low mass density; (4) narrow Brillouin spectral width, $\Gamma_{B}$ (i.e., long phonon lifetime, $\tau_{A}$); and (5) large Pockels photo-elastic constant. Unfortunately, a low density typically suggests a relatively low refractive index, so the density and index tend to mitigate one another with respect to $g_{B}$.

Silica and high silica-content glasses, from which most practical optical fibers are made, are intrinsically low loss, mechanically robust and compatible with other fiber components [51]. Glasses have advantages over semiconductors, semiconductor-doped glasses, and organic materials, as they possess fast response times, negligible linear loss, and small TPA [52]. Linear and nonlinear refractive indices are both attributed to the polarizability and hyperpolarizability of the constituent ions. In conventional silicate glasses, network modifiers such as alkali oxide, increase the number of highly polarizable non-bridging oxygen atoms, thus inducing a moderate increase in nonlinearity [37].

Heavy-metal cations such as lead, bismuth or thallium, are known to impart considerable nonlinear properties to a range of glass formers, for example, silicate, borate and germanate [53,54]. 5- to 10-fold enhancement of the nonlinear Kerr coefficient was demonstrated in lead silicate [55], bismuth silicate [56] and bismuth borate [57,58]. The same increase in Brillouin gain was observed in bismuth silicate [59] and bismuth borate [60]. Other heavy-metal oxide glasses, such as bismuthate [61] and tellurite [62,63], have also shown pronounced Kerr and Brillouin nonlinearities.

The chalcogenide glass family is among the most attractive materials for nonlinear optical applications [64]. Chalcogenides are amorphous semiconductors that contain one or more of the chalcogen elements from group 6a of the periodic table: sulphur, selenium and tellurium, but excluding oxygen, covalently bonded to network formers such as As, Ge, Sb, Ga, Si or P. A broad range of possible glass-forming systems is available, with good resistance to crystallization [65]. Chalcogenides exhibit a high refractive index, pronounced nonlinearities (2–3 orders of magnitude stronger than in silica), a variety of photosensitivity effects, and broad transparency windows from the visible to the middle-infrared wavelength ranges. Well-established techniques of chalcogenide fiber drawing [66] and planar devices fabrication [67], alongside those that are being developed [68,69], pave the way for ever-advancing optical signal processing and sensing technologies. Chalcogenide devices offer among the strongest nonlinearities but also benefit from negligible TPA, compared with silicon.

Silicon is a favourable platform for the realization of passive photonic devices such as low-loss waveguides, cascaded Mach-Zehnder interferometer filters [70], or ring resonators [71]. Its advantages include compatibility with the complementary metal-oxide-semiconductor (CMOS) fabrication process used in microelectronics industry, and high refractive index contrast between silicon core and silicon dioxide cladding, which gives rise to tight optical confinement. However, the realization of active devices in silicon is challenging due to its indirect bandgap and absence of the Pockels effect. Intriguingly, silicon offers an abundance of third-order nonlinear effects but its large TPA coefficient hinders device performance [72]. To circumvent this intrinsic drawback, scientists have had to resort to silicon organic/inorganic hybrids [73,74], slotted waveguide [75], or slow-light approaches based on photonic crystal waveguides [76]. Counterintuitively, interaction between photons and acoustic phonons is markedly weak in those nonlinear waveguides. Nonetheless, the power requirements for SBS can still be reduced substantially by employing high-Q resonators. Slow-light structures and resonators, however, suffer from simultaneous enhancement of competing nonlinear processes. Some enhancement of Brillouin interaction is still possible by exploiting the frequency dependence of the optical density-of-states near the edge of a photonic bandgap [77]. Significant enhancement of the opto-mechanical interaction therefore requires harnessing the full arsenal of optical forces and scattering mechanisms through structural design degrees of freedom [78].

Finally, materials and geometry play an important role as far as sensing applications are concerned. Special care must be taken when designing a point, integrated, or distributed sensor based on BDG. First, the susceptibility of the acoustic velocity and polarization/modal birefringence of waveguides to the measurand of interest is defined by their materials via thermal expansion coefficients, chemical reactivity and so forth, and their geometry via elastic moduli, fiber cladding diameter (or planar waveguide overcladding width) and so forth. Their cross-sensitivity to other environmental parameters is defined by the very same properties. For bonded and embedded sensors, their coating compatibility with adhesives and host materials is also an issue of great practical significance.

## 5. BDG Generation and Interrogation Techniques

It is of practical interest, to leave the framework of CW analysis of BDG creation and interrogation and explore the methods for the localization of: (1) SBS interaction between the pump waves; and (2) probe diffraction from a moving Bragg grating. Over the years, approximate analytical models were developed to accurately describe the spatio-temporal evolution of the acoustic build-up and the probe reflection, allowing the adoption and adaption of various signal processing techniques ubiquitously used in radars, optical reflectometry and so forth.

**Figure 2:**
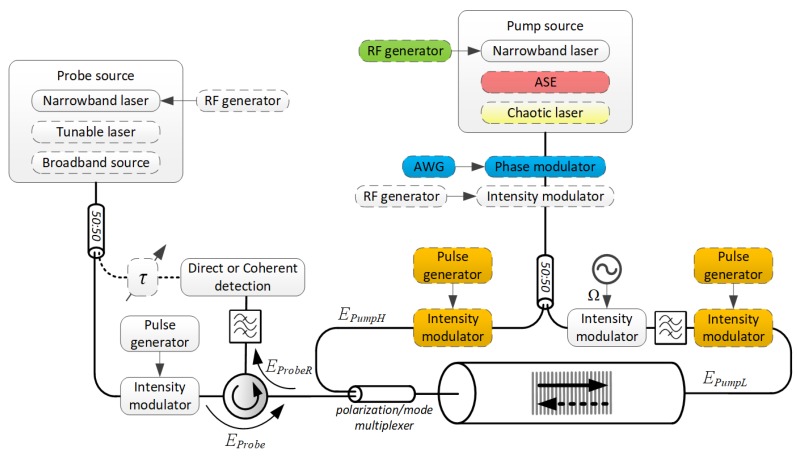
A schematic diagram depicting a complete BDG setup with emphasis on the alternatives for BDG *generation*. Frequently used components are marked with solid lines, while the dashed lines mark the more exotic ones. Colors highlight the components of different BDG *generation* techniques.

Figure 2 shows a schematic diagram of a general-purpose BDG setup, with only principal components included (optical amplifiers, polarization controllers, mode converters/strippers, auxiliary optical filters, lock-in amplification schemes, etc., are excluded for simplicity).

With the help of Figure 2, we will describe in the following sections the alternatives for the generation and characterization of BDGs, alongside their advantages and shortcomings.

### 5.1. Acoustic Build-Up Localization Methods

The location of the BE-FWM interaction in bulk media can be controlled by the angle of incidence and diameter of the intersecting beams. But, those degrees of freedom are absent in translationally symmetric waveguides. The simplest way to obtain localization of BDG in waveguides is through an optical time-gating of counter-propagating pump waves (realized by the components marked in orange color on the diagram of Figure 2).

It is instructive to examine the on-resonance ($\Omega =\Omega_{B}$) solution of the differential equation governing the acoustic wave evolution in time, *t*, along the longitudinal axis of a waveguide, *z*. Here, as well throughout this paper, *PumpH* depletion and *PumpL* amplification, as well as their linear losses, are neglected. The slowly-varying envelopes of the acoustic, Q(z,t), and optical pump waves, $E_{PumpH}$ and $E_{PumpL}$, can be expressed in the form of an exponentially windowed convolution integral [9,29,31]:

(2) $$\;Q\left(z,t\;\right){\;}_{\sim }^{\propto }\;\int_{-\infty }^{t}e^{\frac{-\left(t-t^{\prime }\right)}{2\tau_{A}}}E_{PumpH}\left(t\prime -\frac{z}{c/n_{g}}\right)E_{PumpL}^{*}\left(t^{\prime }+\frac{z-L}{c/n_{g}}\right)dt\prime ,$$

where $n_{g}$ is the group velocity of optical wave packets. At the position and time where the two pump waveforms meet, an acoustic wave starts to build up with a characteristic exponential growth, whose time constant is governed by the phonon lifetime, $\tau_{A}$. As long as the two pump waveforms overlap, an electrostrictive driving force that sustains the BDG is formed. At the time when the two pump waveforms cease to overlap, the acoustic wave starts to decay exponentially. This regime if operation is commonly termed *transient* BDG [17,79]. The length of the transient BDG is dictated by the pump pulses duration. These “stored” pulses can be retrieved later, after a time interval limited by the lifetime of the acoustic excitation [80]. Arbitrary locations along the waveguide can be accessed by properly adjusting the time delay between the two pump pulses.

It is possible to periodically refresh the transient BDG by using a pulse train whose repetition rate, $f_{rep}$, is higher than the acoustic decay rate, $\Gamma_{B}$ [81]. However, the unambiguous distance, $c/2n_{g}f_{rep}$, in such a system becomes impractically small. Furthermore, a tight longitudinal localization of BDG constrains short and high-peak-power pump pulses, and as the duration of the pulses approaches $\tau_{A}$, the acoustic field fails to build up to its full strength and narrow ($\sim \Gamma_{B}$) Lorentzian-shaped BGS [82].

**Figure 3:**
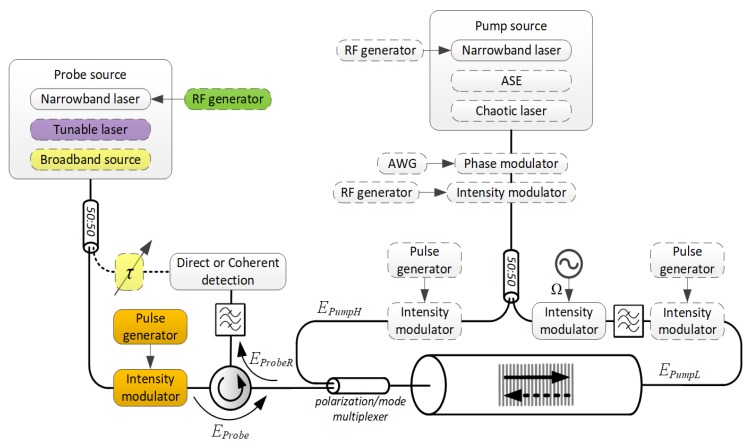
The schematic diagram of Figure 2, highlighting the *interrogation* alternatives of BDGs.

To overcome the aforementioned limitations of transient BDGs, the SBS interaction between the counter-propagating pump waves can be alternatively localized through cross-correlation of their instantaneous frequencies or phases. One of the earliest methods employed synchronous sinusoidal frequency modulation of the pump waves [83] (realized through direct current modulation of the pump laser, marked in green color on the diagram of Figure 2). At point *z* along the waveguide, the instantaneous frequency difference between these two waves is given by [84]:

(3) $$\;\Omega \left(z,t\;\right)=\omega_{PumpH}-\omega_{PumpL}-4\pi \Delta f\sin\left[\pi f_{m}\left(L-2z\right)n_{g}/c\right]\sin\left[\pi f_{m}\left(2t-Ln_{g}/c\right)\right],$$

where Δf is the amplitude of the frequency modulation and $f_{m}$ is its frequency. Interestingly, at those locations obeying $z_{k}=0.5\left(L-kc/n_{g}f_{m}\right)$, known as correlation peaks, the oscillating term in Equation (3) vanishes and $\Omega \left(z_{k},t\right)$ has a time-independent value of $\omega_{PumpH}-\omega_{PumpL}$, as if the BDG is generated by CW pump waves. These correlation peaks, however, have side-lobes which excite BDGs outside the peak zone. Intensity modulation can be used to apodize and suppress those side-lobes such that the localization of the BDG is significantly improved [85]. The center frequency of the BDG at the correlation peaks, $\Omega_{BDG}\left(z_{k},t\right)$, also continuously changes due to the applied frequency modulation. Therefore, it is necessary to apply an identical frequency modulation to the probe wave as well [27] (marked ‘RF generator’ in green color on the diagram of Figure 3). The effective theoretical longitudinal confinement of the BDG that can be achieved with this technique is $\sim c\Gamma_{B}/4\pi^{2}f_{m}\Delta f$ [83]. Hence, tight trade-off exists between the unambiguous distance, $c/2n_{g}f_{m}$, and the spatial extent of the moving Bragg grating. Nevertheless, additional optical time-gating can be applied to remove the ambiguity among the multiple correlation peaks [86].

Another approach to BDG localization, which circumvents the trade-off plaguing the frequency modulation based technique, makes use of a fast phase modulation of the pump waves [28] (realized by the components marked in blue color on the diagram of Figure 2; AWG stands for arbitrary waveform generator). When the pumps modulation is driven by a sequence $c_{n}$ of unity magnitude and a symbol duration T, $E_{PumpH}\left(t,0\right)=E_{PumpL}\left(t,L\right)=\underset{n}{\sum }c_{n}\mathrm{rect}\left[\left(t-nT\right)/T\right]\equiv A\left(t\right)$, subject to discretization of the waveguide into $cT/2n_{g}$ long bins, Equation (2) can be rewritten in the form of an exponentially windowed autocorrelation function with a memory of $N_{0}\equiv \mathrm{round}\left(2\tau_{A}/T\right)$ [87]:

(4) $$\;Q\left(z,t\;\right){\;}_{\sim }^{\propto }\;\overset{n_{0}\left(t,z\right)-1}{\underset{n=-\infty }{\sum }}e^{\frac{-\left(n_{0}-n\right)}{N_{0}}}c_{n}c_{n-l_{z}}^{*}.$$

Here, $n_{0}\left(t,z\right)\equiv \mathrm{round}\left[\left(t-zn_{g}/c\right)/T\right]$ is the bit appearing in the *PumpH* waveform at position *z* and time *t*, and $l_{z}\equiv \mathrm{round}\left[\left(2z-L\right)n_{g}/cT\right]$ is the normalized, position-dependent lag between the sequences. If the random process A(t) is ergodic and stationary in the wide sense [88], and fluctuates much faster than the acoustic lifetime ($T\ll \tau_{A}$), following the initial buildup ($t\gg \tau_{A}$), a correlation peak of constant magnitude is established where $l_{z}=0$, with a spatial extent of $\sim cT/2n_{g}$ (assuming the autocorrelation $A\left(t\right)A^{*}\left(t\right)$ is a delta function). The stationary point can be moved along the waveguide by controlling the delay between the pumps fed into opposite ends of the waveguide.

The first experimental demonstration of this principle relied on the phase modulation of the two pump waves by a common pseudo-random bit sequence (PRBS) [29]. Since the spatial extent of the sustained BDG is governed by the spatial extent of the autocorrelation function, non-zero side-lobes of PRBS drive the buildup of spurious BDGs at locations other than the location of interest (i.e., the correlation peak at $l_{z}=0$). In subsequent publications, the PRBS was replaced with perfect Golomb codes, having a periodic correlation with theoretically zero side-lobes [87]. A significant reduction in the off-peak reflectivity was respectively demonstrated [12].

Phase modulation can be replaced altogether by an amplified spontaneous emission (ASE) optical source (marked in red color on the diagram of Figure 2). BDG confinement to a single and narrow correlation peak of 4 mm was demonstrated with an ASE source filtered to a bandwidth of 25 GHz [89]. Similarly, in principle, a chaotic laser can also be used to obtain narrow correlation peaks (marked in yellow color on the diagram of Figure 2). A recent demonstration employed an innovative modification of the external cavity feedback scheme of a semiconductor laser [90], which allowed the suppression of characteristic time-delay signatures and inhibition of the detrimental periodic correlation peaks [91].

It is important to remember that spontaneous Brillouin scattering from thermally initiated acoustic phonons always accompanies the processes of BDG generation and interrogation [92,93]. This noisy emission, which cannot be separated from the signal of interest, may have little practical impact on BDG operation in silica fibers, but is very much relevant to BDG operation in devices with giant Brillouin gains.

### 5.2. Grating Readout Methods

Under the undepleted pump and probe approximation, as well as other conditions applicable to BDG operation in short silica fibers, the corresponding impulse response of the transfer function of Equation (1) is given by [94]:

(5) $$\;h(t)\;{\;}_{\sim }^{\propto }\;\frac{\Gamma_{B}\cdot \mathrm{rect}\left(ct/2nL\right)}{\sqrt{4{{\Delta \Omega }_{B}}^{2}\left(ct/2n\right)+\Gamma_{B}^{2}}}\cdot e^{iarctg\left[2{\Delta \Omega }_{B}\left(ct/2n\right)/\Gamma_{B}\right]}\cdot e^{i{\Delta \Omega }_{BDG}\left(ct/2n\right)t}\cdot e^{-i\left(\omega_{Probe}-\Omega \right)t}.$$

The leading ratio in Equation (5) is the amplitude of the reflection. It is a function of the longitudinally-distance-dependent mismatch between the pumps frequency difference, Ω, and the BFS, $\Omega_{B}\left(ct/2n\right)$. The next phase factor represents the dependence of the phase of the probe reflection from the receding grating also on ${\Delta \Omega }_{B}\left(ct/2n\right)$. The third phase factor originates from a mismatch between the incoming probe frequency and the resonant frequency of the moving Bragg grating, ${\Delta \Omega }_{BDG}\left(ct/2n\right)$. This phase too is distance dependent due to the longitudinal variations of the fiber birefringence, Δn(ct/2n). In general, ${\Delta \Omega }_{B}$ and ${\Delta \Omega }_{BDG}$ are nonzero and vary along the fiber. Thus, h(t) is complex-valued, having distance-dependent magnitude and phase. Equation (5) ends with the phasor of the Stokes reflection with frequency $\omega_{Probe}-\Omega \equiv \omega_{ProbeR}$. The BDG response to the incoming probe, $E_{Probe}\left(t\right)\;e^{-i\omega_{Probe}t}$, is thus a convolution integral: $E_{Probe}\left(t\right)⨂h\left(t\right)$.

A distributed measurement of BDG properties can be accomplished in several different ways, each with its shortcomings. The most straightforward choice is to send a *Probe* pulse into the waveguide where a BDG was established along its entire length by CW pumps (the *Probe* pulse is realized by the components marked in orange color on the diagram of Figure 3). This method is actually what most BDG applications use, albeit at increased risk of pump depletion. When the BDG is of transient nature [13,17,19], a proper synchronization of the *Probe* with *PumpH* is required, in order to fall within the time interval governed by the phonon lifetime. Localized and stationary BDGs, on the other hand, can simply be interrogated by a CW probe (note that in this case, the grating length L in Equation (5) effectively represents the region where the BDG was confined, rather than the length of the entire waveguide). The most prevalent form of acquisition of probe reflections is direct detection. An estimate of the local BFS, $\Omega_{B}$, or the BDG resonant frequency, $\Omega_{BDG}$, can be obtained from power spectrum measurements. The phase of the reflections also plays an important role in BDG characterization. Interferometric measurements of temporally [95] or spectrally [24] spaced reflections have demonstrated superior sensitivity.

The fact that the creation of BDG and its interrogation are separated, and that its reflections can be treated as a linear system (as manifested in Equation (5)), allows for the employment of long-known radar and optical signal processing techniques for the detection and processing of distributed reflections.

In time-domain techniques, the spatial resolution is dictated by the probe pulse duration while the signal-to-noise ratio depends on its peak-power. Unfortunately, in the commonly used silica fibers, the reflectivity of the probing signal is extremely low. The launched power, nevertheless, can be kept within practical limits while providing the required spatial resolution, by means of pulse compression techniques [96], in which the energy in a long, low-power, specially designed probe waveform, is integrated through matched (or mismatched) filtering, into a virtual short pulse of high power (the *Probe* pulse generator of Figure 3 is replaced with an AWG). Being a coherent sum of multiple reflections, the reflected probe now contains interference terms that cannot be ignored, so the commonly employed direct detection cannot be used for proper pulse compression. Coherent detection, while more complex and expensive to implement, has proven instrumental in boosting the performance of BDG-based reflectometry [23], that otherwise requires time-consuming trace-averaging, which hinders its measurement speed. The low reflectivity of BDGs can also be partially mitigated by heterodyne detection [97,98].

Swept-Wavelength Interferometry (SWI) constitutes a more natural tool for the comprehensive characterization of BDG complex transfer functions (featuring both amplitude $\left|H\left({\Delta \Omega }_{B},{\Delta \Omega }_{BDG}\right)\right|$ and phase $∠H\left({\Delta \Omega }_{B},{\Delta \Omega }_{BDG}\right)$). The principle of operation behind SWI is the frequency modulated continuous wave (FMCW) interference (beating), which was originally investigated in electric radar systems. SWI comprises a tunable laser source whose frequency is swept continuously (marked in purple color on the diagram of Figure 3), and a Mach-Zehnder interferometer. Light from the source is split and launched into the measurement and reference arms of the interferometer, and recombined at the photodetector. *Probe* light in the measurement arm is upshifted by Ω (the *Probe* pulse generator of Figure 3 is replaced with an RF generator to that end) and fed through an optical circulator into the waveguide where a BDG was established. If the optical frequency of the tunable laser source is modulated at a constant rate, the frequency difference between the light propagating in the reference arm and the light reflected from the BDG, *ProbeR*, is proportional to the optical path difference between them, producing time-varying interference fringes upon arrival at the detector. For fully distributed reflections, the beat signal will contain a continuous distribution of frequencies that can be processed into the time domain using the Fourier transform, and subsequently, a map of reflections as a function of time (length) can be constructed. The spatial resolution of SWI is determined by the frequency scan range of the tunable laser source, while its coherence length dictates the maximum measurement range of the technique. SWI simultaneously achieves centimeter-scale spatial resolution and high signal-to-noise ratio [39,99,100], as it resolves the fundamental trade-off associated with pulsed interrogation of BDGs, where both the spatial resolution and the transmitted energy depend upon the pulse duration.

Finally, Optical Low Coherence Reflectometry (OLCR), realized by the components marked in yellow color on the diagram of Figure 3, whose spatial resolution is determined by the spectral width of a broadband light source, was shown to resolve features down to 100 µm on a BDG reflectogram [40,101]. Here, as well, the *Probe* light is upshifted by Ω before entering the interaction medium.

## 6. BDG Applications

Once the BDG was established, sending the *Probe* into the waveguide may serve two principal purposes. First, if the BDG position, amplitude and phase are known, then the *Probe* is simply a test signal to be processed by the function that the BDG was intended to realize. The other case is when the properties of the BDG are not known in advance (it is working as a sensor of some measurand) and the purpose of sending the *Probe* into the waveguide is to study those properties.

### 6.1. All-Optical Signal Processing

The main driving force for integrated photonics research is data interconnects, which require broad rates, high density and low power dissipation. Optical signal processing can potentially compete with electronics in very high-speed systems that provide only limited functions [102]. All-optical time differentiation, time integration and true time reversal (TTR) were demonstrated in [9], the latter having no similar counterpart in electronics. Practically all signal processing functionalities that employ Bragg gratings [103], can be realized with the BDG platform, with the obvious advantage that BDGs can be dynamically reconfigured. Experimentally validated BDG-based microwave photonic reconfigurable filters [10], phase-shifted BDGs [104], tunable optical delay lines [11,12] and isolators [50], alongside the theoretically predicted pulse compression [14] and frequency combs [15], supplement the photonic building blocks inventory. Coherent acousto-optic memory [13] with somewhat limited power efficiency and storage time, can be employed in optical circuitry, for a complete toolset.

### 6.2. Reflectometers and Sensors

Among the more interesting measurement apparatuses that employ the BE-FWM effect, is the ultrahigh-resolution optical spectrometry [16]. By operating a BDG in a 400 m SMF, a 4 fm (0.5 MHz) spectral resolution is achieved, with extended wavelength coverage up to C- and L-bands using a commercially available tunable laser.

Distributed measurement of birefringence [17,95] and intermodal beat length [18,26], is the most natural application of BDG-based reflectometry, since by simply measuring the resonant frequency of the BDG readout along the fiber, ${\Delta \Omega }_{BDG}\left(z\right)$, the longitudinal variations of the fiber birefringence, Δn(z), can be deduced, an accuracy of 10^−8^ refractive index units with a spatial resolution of 30 cm being the state of the art.

Truly distributed multi-parameter fiber sensing is probably the most practical application of BDGs in optical fibers, as evidenced by the exhaustive review of [105]. The longitudinal acoustic velocity in the fiber, $V_{a}$, upon which the slow axis BFS depends, is proportional to the axial strain or/and temperature change [106]. In line with the justified assumption that the interrogating *Probe* pulse does not deplete the acoustic grating (whose intensity is rather solely determined by the pumps powers, their frequency detuning and the properties of the interaction medium), the *Probe* reflectivity appears to scale proportionally with the grating intensity. Distributed BDG sensing is accomplished by scanning the frequency difference between the writing pumps, Ω, and for each value of their frequency difference, recording the *Probe* pulse reflectivity. This process distributedly characterizes the narrow BGS of the interacting pumps, resulting in the determination of the measurand-dependent value of the slow axis BGS peak, that is, its BFS along the fiber, $\Omega_{B}\left(z\right)$.

In a sense, the BDG sensing imitates the classical BOTDA [107], albeit obtaining the BGS from the intensity of the reflected *Probe* rather than from the gain of the signal wave. Due to the separation of the creation of the acoustic wave from its interrogation, the spatial resolution of the BDG sensing, which is determined by the width of the *Probe* pulse, can be arbitrarily high. Practically, the pulse width can be as narrow as allowed by signal-to-noise considerations. Sub-centimeter spatial resolution was demonstrated in [20].

Standard BDG sensing techniques disregard the fact that the phase matching between the BDG and the incoming *Probe* wave, $\Omega_{BDG}\left(z\right)$, may vary along the fiber. Recently, it was noted that while the BFS linearly increases with either fiber elongation or heating, the birefringence, Δn(z), upon which the phase matching between the *Probe* and BDG depends, responds differently. In the case of Panda-type PMF, for instance, when the temperature increases the residual stress between the B_2_O_3_-doped-silica stress-applying parts and the pure-silica cladding, formed during the fiber drawing process, weakens and the birefringence decreases. In contrast, when an axial strain is applied, additional stress is generated because the stress-applying parts and the cladding contract in the lateral direction differently due to their different Poisson’s ratios, and the birefringence increases. The distributed birefringence profile can be deduced from the measurement of the BDG resonant frequency, $\Omega_{BDG}\left(z\right)$, along the fiber. Harnessing this second degree of freedom allowed for a complete discrimination between strain and temperature in PMFs [108].

Relying on the same characterization method, other environmental parameters may also be measured with specialty fibers. Transverse load was measured in an elliptical-core PMF, with a measurement accuracy of 0.8∙10^−3^ N/mm [21]. Hydrostatic pressure measurement was experimentally demonstrated in a PCF [22], with a maximum measurement error of 0.03 MPa. Both demonstrations achieved a spatial resolution of 20 cm.

To extend the time-domain BDG-based sensing to the dynamic regime, several protocols were devised. First, rather than scanning a wide range of frequencies, the slope of BDG reflectance was employed, directly translating fiber deformations to *Probe* amplitude changes allowing for substantial increase in the effective sampling rate of the sensor [33]. Second, pulse compression techniques were used to further improve the measurement precision [23]. And third, the interference of simultaneous Stokes and anti-Stokes BDG reflections enabled the measurement of Brillouin induced phase-shift (the first phase factor in Equation (5)) [24]. The technique was found to be largely immune to variations in laser optical power and fiber bend losses, which significantly reduces the cross-sensitivity of slope-assisted systems. Working on the slope of BDG phase-shift together with efficient coding, potentially offers one of the best combinations of speed (limited by time of flight: e.g., 1 MHz sampling rate for 100 m) and spatial resolution (limited by symbol duration, down to millimeters) for dynamic strain measurements. Those state-of-the-art systems were used to measure strain oscillations approaching ultrasonic frequencies (the tested vibration frequency of 10 kHz in [94] was limited by the speed of the mechanical shaker) without sacrificing spatial resolution (the 2 cm resolution in [94,109] was limited by the bandwidth of the available photoreceiver).

## 7. Conclusions and Perspectives

This review attempted to provide a historical perspective on Brillouin dynamic gratings. A comprehensive survey of the past and present platforms to host BDG interactions was carried out. The application of the BE-FWM effect to the synthesis of dynamically reconfigurable gratings, offers great potential for a variety of signal processing elements for photonic circuits. This capability has thus far been conceptually demonstrated in optical fibers, albeit requiring high operating power and with limited efficiency. From a practical standpoint, in order to carry over this concept to integrated photonic devices, those elements must be made compact, efficient and operate at low power. Recently emerging technologies of nonlinear planar waveguides in chalcogenide glasses and silicon [110] may accomplish just that. Exciting challenges await researchers while trying to harness the unique features of BDGs in waveguides having remarkably strong light and sound interactions.

Conversely, BDG-based reflectometry and sensing may evolve both as a fiber optic and photonic device technology. The combination of high speed and spatial resolution may prove instrumental in high-end fiber optic sensors applications, such as monitoring the propagation of mechanical waves [111] and structural damage identification [112]. The monitoring of smaller structures and faster vibrations naturally calls for higher spatial resolution which BDG-based sensing possesses. Also, faster dynamic phenomena, such as acoustic emissions, can be potentially measured [113,114]. BDG-based sensors must also be evaluated for compliance with guidelines and standards of fiber optic sensors [115]. In integrated devices, the characterization of local optical and mechanical properties of a waveguide is no less important. Many sensing schemes can also be thought of.

This review has used the term Brillouin-Enhanced Four-Wave-Mixing (BE-FWM) to exclusively describe the phenomenon of Brillouin dynamic gratings (BDGs). In a broader literal sense, BE-FWM is also relevant to other Brillouin enhancement effects in FWM interactions, such as described in [116,117], which do not involve BDGs and, consequently, are not covered in the review.
